# Burnout and Quality of Life Among Oncology Palliative Care Professionals: An Exploratory Study

**DOI:** 10.3390/healthcare14193239

**Published:** 2026-10-01

**Authors:** José Manuel Barbosa Teixeira, Tiago Paredes, Ana Almeida Ribeiro, Sofia Nunes, Francisca Rego, Guilhermina Rego, Rui Nunes

**Affiliations:** 1 Faculty of Medicine, University of Porto, 4200-319 Porto, Portugal; josemanuel.geral@gmail.com (J.M.B.T.); asnunes@med.up.pt (S.N.); mrego@med.up.pt (F.R.); guilherminarego@med.up.pt (G.R.); ruinunes@med.up.pt (R.N.); 2 Alto Ave Local Health Unit, Rua dos Cutileiros, Creixomil, 4835-044 Guimarães, Portugal; 3 Faculty of Psychology and Education Sciences, University of Coimbra, 3000-115 Coimbra, Portugal; tiago_paredes@live.com; 4 Higher School of Health of Viseu, Polytechnic University of Viseu, 3500-843 Viseu, Portugal; 5 Health Sciences Research Unit, Nursing (UICISA:E), Nursing School of Coimbra, 3004-011 Coimbra, Portugal

**Keywords:** palliative care, oncology, burnout, quality of life, physicians, nurses, healthcare professionals

## Abstract

**Highlights:**

**What are the main findings?**

- No statistically significant multivariate differences in burnout or quality of life were detected across oncology palliative care, intensive care, and emergency care in this small exploratory sample.
- A secondary, unadjusted comparison identified a nominally significant difference in emotional exhaustion between oncology palliative care professionals and a heterogeneous reference group; this finding is hypothesis-generating.

**What are the implications of the main findings?**

- Non-significant findings in a small, unequal convenience sample do not establish similarity or equivalence between clinical settings.
- Larger multicentre studies with well-characterized professional groups and working conditions are needed to assess the exploratory emotional-exhaustion finding.

**Abstract:**

**Background/Objectives**: Oncology palliative care involves sustained engagement with suffering, loss, complex communication, and end-of-life decisions. This exploratory study compared burnout and quality of life among professionals working in oncology palliative care, intensive care, and emergency care, and secondarily with a heterogeneous reference group. **Methods**: A cross-sectional convenience sample included 68 healthcare professionals from two hospital units within the same Portuguese public hospital organization: oncology palliative care (*N* = 22), intensive care (*N* = 15), emergency care (*N* = 9), and other healthcare professionals (*N* = 22). Burnout and quality of life were assessed using the Maslach Burnout Inventory—Human Services Survey and WHOQOL-BREF, respectively. Analyses used complete cases for each multivariate outcome set. **Results**: No statistically significant multivariate differences were detected across the three clinical groups in burnout (Pillai’s Trace = 0.187; F(6, 84) = 1.442; *p* = 0.208) or quality of life (Pillai’s Trace = 0.146; F(10, 78) = 0.613; *p* = 0.798). In the secondary burnout comparison (*n* = 38), emotional exhaustion was higher in oncology palliative care (mean difference = 5.78; 95% CI 0.37–11.19; unadjusted *p* = 0.037; partial η^2^ = 0.115). No multivariate quality-of-life difference was detected in the secondary comparison (*n* = 39; *p* = 0.387). **Conclusions**: The non-significant findings do not establish equivalence between clinical settings. The nominal emotional-exhaustion difference is hypothesis-generating and requires replication. Small, unequal groups, missing data, multiple testing, and limited occupational characterization constrain precision and interpretation.

## 1. Introduction

Oncology palliative care requires healthcare professionals to combine complex clinical care with sustained emotional presence. Palliative care aims to prevent and relieve suffering while supporting the quality of life of people living with life-threatening illness and their families [1]. In oncology, this involves not only symptom management but also communication about prognosis and goals of care, support for patients and families, and decision-making as illness progresses. Because therapeutic relationships may extend across prolonged and uncertain disease trajectories, professionals are repeatedly exposed to deterioration, suffering, loss, and death. This work can be deeply meaningful, but it may also place considerable demands on their emotional and psychological resources.

Burnout represents one important dimension of professional wellbeing. It is commonly described through emotional exhaustion, depersonalization, and reduced personal accomplishment [2], and is recognized by the World Health Organization as an occupational phenomenon resulting from chronic workplace stress that has not been successfully managed [3]. Burnout develops within the interaction between professionals and their working environments, including workload, staffing, role clarity, recognition, leadership, team functioning, and access to support. Its relevance extends beyond the individual professional, as healthcare-worker wellbeing is closely connected to workforce sustainability, care quality, and patient safety [4,5]. Quality of life provides a complementary perspective by encompassing physical health, psychological wellbeing, social relationships, and the broader living environment.

Although palliative care professionals are often considered particularly vulnerable to burnout, the available evidence does not demonstrate a consistent pattern. Reported estimates vary considerably according to profession, clinical context, sample characteristics, measurement approach, and organizational conditions [6]. Studies conducted in Portugal have also identified concerns regarding burnout and quality of life among oncology and palliative care professionals [7,8]. However, much of the existing research has examined individual services or compared professionals within oncology and palliative-care environments. It therefore remains unclear whether oncology palliative care professionals experience a distinct pattern of burnout and quality of life when compared directly with professionals working in other demanding healthcare settings.

Intensive and emergency care provide clinically relevant comparators because they are not low-stress environments. Professionals working in these settings also face mortality, acute deterioration, ethically complex decisions, time pressure, interruptions, and demanding shift patterns [9,10]. Nevertheless, the nature of these demands differs across settings. Oncology palliative care is more often characterized by sustained therapeutic relationships and cumulative exposure to progressive illness, whereas intensive and emergency care involve greater acuity, unpredictability, and compressed decision-making. Comparing these contexts can therefore help clarify whether the wellbeing profile observed in oncology palliative care reflects its sustained relational demands or forms part of the broader burden associated with high-intensity healthcare. A heterogeneous reference group of other healthcare professionals from the same institutions provides an additional occupational perspective beyond these three clinical settings.

Professional wellbeing may also be shaped by the organizational and psychosocial context in which care is delivered. Supportive leadership, recognition, collegial relationships, working conditions, and professional satisfaction have been associated with burnout and wellbeing among healthcare professionals [11,12,13]. Coping and communication resources may also be relevant to emotionally demanding practice [14,15]. These factors provide context for interpreting differences between professional groups; the present report focuses on comparisons of burnout and quality of life rather than associations with satisfaction or coping.

Accordingly, the primary objective of this exploratory study was to compare burnout dimensions and quality-of-life outcomes among physicians and nurses working in oncology palliative care, intensive care, and emergency care. A secondary objective was to compare oncology palliative care professionals with a heterogeneous reference group of other healthcare professionals. Both comparisons were exploratory and were not designed to establish equivalence between groups.

## 2. Materials and Methods

### 2.1. Study Design and Setting

This quantitative, exploratory, cross-sectional study was conducted across two hospital units belonging to the same Portuguese public hospital organization in Northern Portugal. Participants were recruited from both institutions and comprised four groups: oncology palliative care, intensive care, emergency care, and a heterogeneous reference group of other healthcare professionals. The primary comparison concerned the three clinical groups; the comparison with the reference group was secondary. Study reporting was informed by the STROBE recommendations for cross-sectional observational studies [16]. The completed STROBE checklist is provided in Supplementary File.

### 2.2. Participants, Eligibility, and Recruitment

A non-probability convenience sample of 68 healthcare professionals was included, comprising those working in oncology palliative care (*N* = 22), intensive care (*N* = 15), emergency care (*N* = 9), and a heterogeneous reference group of other healthcare professionals (*N* = 22). The three clinical groups comprised physicians and nurses working in the corresponding services and together included 46 participants. Throughout this report, N denotes the total recruited group size, and n denotes the number of valid responses or complete cases in a specified analysis.

Eligible participants were adult healthcare professionals working in one of the participating institutions who agreed to participate and provided informed consent. Recruitment followed a convenience-sampling approach. Participation was voluntary, and the resulting sample should not be interpreted as a census or a probability sample of the eligible hospital workforce.

### 2.3. Data Collection and Ethical Considerations

Data were collected between December 2025 and February 2026 using a self-administered assessment protocol. The present report uses the sociodemographic and professional information and the burnout and quality-of-life measures described below. Participants received written information about the study purpose and procedures, voluntary participation, confidentiality, and anonymity. Written informed consent was obtained before questionnaire completion. The study was conducted in accordance with the Declaration of Helsinki, and prior institutional authorization was obtained. The documented ethics approval is reported in the Institutional Review Board Statement.

### 2.4. Measures

#### 2.4.1. Sociodemographic and Professional Questionnaire

The sociodemographic and professional questionnaire collected information on age, sex, marital status, educational and postgraduate qualifications, profession, clinical setting, and selected aspects of professional experience.

#### 2.4.2. Maslach Burnout Inventory—Human Services Survey

Burnout was assessed using the Maslach Burnout Inventory—Human Services Survey (MBI-HSS), a 22-item self-report instrument comprising three dimensions: emotional exhaustion (nine items), depersonalization (five items), and personal accomplishment (eight items) [17]. Items are rated from 0 (never) to 6 (every day). Higher emotional-exhaustion and depersonalization scores represent less favourable outcomes, whereas lower personal-accomplishment scores indicate a less favourable outcome. The Portuguese adaptation described by Melo et al. was used [18]. Authorization for use of the MBI-HSS was obtained before data collection.

The MBI-HSS was administered to all four groups, and continuous subscale scores were used in the analyses. Emotional exhaustion, depersonalization, and personal accomplishment were retained as separate outcomes rather than combined into a total burnout score. Burnout categories were not applied because their interpretation depends on the reference population and applicable normative criteria.

#### 2.4.3. WHOQOL-BREF

Quality of life was assessed using the Portuguese version of the WHOQOL-BREF [19]. This 26-item self-report instrument comprises four domains—physical health, psychological wellbeing, social relationships, and environment—and two general items addressing overall quality of life and satisfaction with health. Domain scores were transformed to a 0–100 scale, with higher values indicating better perceived quality of life. The instrument was administered to all four groups. The reported analyses included the four domain scores and a combined general quality-of-life/health outcome based on the two general items. This general outcome is distinct from the four domain scores. Study-specific internal-consistency estimates were not calculated and are therefore not reported.

### 2.5. Sample Size and Statistical Analysis

No a priori sample-size calculation was performed. The available sample was small and unequally distributed, particularly in emergency care (*N* = 9). The study was therefore treated as exploratory. Estimates may be imprecise and unstable, and non-significant tests cannot exclude clinically relevant differences or establish equivalence between groups.

Statistical analyses were conducted using IBM SPSS Statistics, version 20.0 (IBM Corp., Armonk, NY, USA). Continuous variables were summarized using means and standard deviations (SDs) or standard errors (SEs), as specified in each table. Categorical variables were described using absolute and relative frequencies with explicit valid-response denominators. SD describes between-participant variability, whereas SE describes uncertainty in a mean estimate.

Analyses were structured in two stages. First, burnout and quality-of-life outcomes were compared across oncology palliative care, intensive care, and emergency care using one-way multivariate analysis of variance (MANOVA). Pillai’s Trace was selected for the primary three-group analyses because of its relative robustness to moderate departures from multivariate normality and homogeneity of covariance matrices in the presence of unequal group sizes. The three MBI-HSS dimensions and the five reported WHOQOL-BREF outcomes were analysed as separate multivariate outcome sets.

Second, oncology palliative care professionals were compared with the heterogeneous reference group using two-group MANOVA, reported through Hotelling’s Trace. Corresponding univariate results were reported with exact *p*-values and partial eta-squared effect sizes; secondary comparisons also included mean differences and 95% confidence intervals. These were unadjusted group comparisons, and the estimates should not be interpreted as effects of clinical setting independent of professional category, age, institution, or working conditions.

The nominal significance level was 0.05. No formal adjustment for multiple testing was applied. Consequently, *p*-values and confidence intervals are unadjusted, and a nominally significant secondary result was interpreted as hypothesis-generating rather than confirmatory. Effect sizes describe the observed sample estimates and do not resolve uncertainty arising from small groups, missing data, or multiple comparisons.

### 2.6. Missing Data

Descriptive statistics used the available valid responses for each variable. Multivariate analyses included only participants with complete scores for all outcomes in the corresponding outcome set. Thus, the number analysed could differ between the MBI-HSS and WHOQOL-BREF models and between primary and secondary comparisons. The secondary models included 38 complete cases for the MBI-HSS and 39 for the WHOQOL-BREF, out of 44 participants in the two groups combined. No imputation of missing data was performed.

## 3. Results

### 3.1. Participant Characteristics

The study included 68 healthcare professionals: 22 in oncology palliative care, 15 in intensive care, 9 in emergency care, and 22 in the heterogeneous reference group. Professional-category information was available for 66 participants: 49 (74.2%) were nurses and 17 (25.8%) were physicians. Professional category was missing for one participant in oncology palliative care and one in intensive care. The heterogeneous reference group comprised 15 nurses (68.2%) and 7 physicians (31.8%). Participants in this group had a mean age of 34.61 years (SD = 7.85); 18 (81.8%) were women and 13 (59.1%) were married or cohabiting. Other valid-response denominators varied by characteristic, as shown in Table 1.

### 3.2. Burnout Across Professional Settings

No statistically significant multivariate difference in MBI-HSS dimensions was detected across the three clinical groups (Pillai’s Trace = 0.187; F(6, 84) = 1.442; *p* = 0.208; partial η^2^ = 0.093). None of the corresponding univariate tests reached nominal statistical significance. Emotional exhaustion was numerically higher in oncology palliative care, but the across-group test was not significant (*p* = 0.140). These findings do not establish equivalence between settings (Table 2).

### 3.3. Quality of Life Across Professional Settings

No statistically significant multivariate difference in WHOQOL-BREF outcomes was detected across the three clinical groups (Pillai’s Trace = 0.146; F(10, 78) = 0.613; *p* = 0.798; partial η^2^ = 0.073). None of the corresponding univariate tests reached nominal statistical significance (all *p* ≥ 0.442). Estimates for the four domain scores and the combined general outcome are presented in Table 3. The non-significant tests do not demonstrate equivalent quality of life across settings.

### 3.4. Secondary Comparisons with Other Healthcare Professionals

#### 3.4.1. MBI-HSS Comparison

The secondary MBI-HSS comparison included 38 complete cases from the 44 participants in the two groups combined. The multivariate test reached nominal statistical significance (Hotelling’s Trace = 0.260; F(3, 34) = 2.942; *p* = 0.047; partial η^2^ = 0.206). Emotional exhaustion was higher in oncology palliative care (mean difference = 5.78; 95% CI 0.37–11.19; unadjusted *p* = 0.037; partial η^2^ = 0.115). Personal accomplishment and depersonalization did not reach nominal statistical significance. Given multiple unadjusted comparisons and uncertainty in the estimates, the emotional-exhaustion result is hypothesis-generating (Table 4).

#### 3.4.2. WHOQOL-BREF Comparison

The secondary WHOQOL-BREF comparison included 39 complete cases from the 44 participants in the two groups combined. No statistically significant multivariate difference was detected (Hotelling’s Trace = 0.164; F(5, 33) = 1.085; *p* = 0.387; partial η^2^ = 0.141). None of the corresponding univariate tests reached nominal statistical significance, and all confidence intervals for mean differences included zero. These results do not establish equivalence (Table 5).

## 4. Discussion

This exploratory study did not detect statistically significant multivariate differences in burnout or quality of life among professionals working in oncology palliative care, intensive care, and emergency care. A secondary comparison with a heterogeneous reference group identified a nominally significant difference in emotional exhaustion. These two findings require different qualifications: the non-significant primary tests do not establish equivalence, and the secondary result does not establish a setting-specific vulnerability. The small, unequal groups and multiple unadjusted comparisons limit the strength of both interpretations.

The palliative-care literature reports substantial variation in burnout according to professional composition, working conditions, and measurement approach [6]. In a Portuguese study of 34 professionals working in oncology palliative care, Gonçalves and Gaudêncio reported mean emotional exhaustion of 22.2 (SD 13.3), with numerically higher exhaustion among physicians and nurses than among other professional groups [7]. This provides a relevant descriptive comparison with the present oncology palliative care mean of 19.77, but differences in sample composition and study design preclude an inferential comparison between the studies. The occupational composition of the reference group is therefore particularly important to interpretation.

Additional Portuguese evidence illustrates why clinical setting alone is an insufficient explanation. A study of 75 palliative-care physicians using the Copenhagen Burnout Inventory reported high work-related burnout in 52% and high patient-related burnout in 21%; exclusive dedication to palliative care and the type of service were associated with burnout outcomes [20]. These findings highlight occupational context but cannot be directly compared with MBI-HSS scores because the instruments assess different dimensions and the study concerned the COVID-19 period. Other Portuguese studies likewise support examining professional and organizational characteristics rather than assuming a uniform palliative-care profile [8,21,22].

Intensive and emergency care are demanding comparator settings rather than low-stress controls. Evidence from these environments identifies workload, acuity, mortality, and organizational conditions as relevant to burnout [9,10]. However, the present non-significant comparisons cannot determine whether the occupational burden is shared equally across settings or differs in ways that the available sample could not detect. In particular, the emergency-care group included only nine participants. Numerical patterns should consequently be interpreted as exploratory estimates, not as evidence of similar wellbeing.

The secondary emotional-exhaustion difference warrants investigation because oncology palliative care can involve sustained therapeutic relationships and repeated encounters with suffering and death. Prior research suggests that such exposure may be associated with professional wellbeing, with effects shaped by individual, relational, and organizational conditions [6,23]. In the present study, however, workload, staffing, shift patterns, exposure to patient death, team functioning, and access to supervision were not characterized sufficiently to test these explanations. Cumulative relational demands remain a possible mechanism rather than a demonstrated explanation of the observed difference.

Clinical setting was also potentially confounded with professional category and age. Among participants with valid professional-category information, physicians represented 44.4% of the emergency-care group, compared with 19.0% in oncology palliative care and 14.3% in intensive care. The emergency-care group also had a higher mean age among valid responses. These compositional differences, and differences between institutions, may have influenced the unadjusted comparisons. Limited characterization of the heterogeneous reference group further prevents attributing the emotional-exhaustion estimate to the palliative-care setting independently of professional role or working conditions [11,22,24].

The separate MBI-HSS dimensions should be retained in interpretation. Emotional exhaustion, depersonalization, and personal accomplishment are conceptually distinct [2], and oncology nursing research has examined their relationships with communication and the working environment [15]. A nominal difference in one dimension does not demonstrate globally greater burnout. Equally, non-significant differences in the remaining dimensions do not demonstrate that those dimensions are unaffected. The present findings support dimensional reporting while remaining insufficient to establish a distinctive professional profile.

Quality of life is broader than occupational burnout, encompassing physical health, psychological wellbeing, social relationships, and environmental conditions [25,26]. Gonçalves and Gaudêncio did not identify statistically significant associations between burnout dimensions and WHOQOL-BREF domains in their preliminary oncology palliative-care sample [7]. Such findings illustrate the importance of distinguishing the constructs, but neither that study nor the present between-group tests establish independence, resilience, or protection of global wellbeing. Studies of professional quality of life also discuss positive and negative occupational experiences [27]; these constructs should not be treated as interchangeable with the general quality of life assessed by WHOQOL-BREF.

Organizational assessment remains relevant to professional wellbeing. Workload, staffing, autonomy, leadership, recognition, and access to support have been associated with burnout in healthcare [11,24,28]. Confidential wellbeing assessment, reflective team discussion, supervision, and review of work organization are potential areas for further evaluation. The present study assessed no intervention and cannot establish the effectiveness of these measures. Evidence from organizational interventions suggests that outcomes depend on intervention design, implementation, and context [29].

### 4.1. Strengths and Limitations

A strength of this study is its comparison of oncology palliative care with two other demanding clinical settings within the same institutional framework, while retaining the separate burnout dimensions and quality-of-life outcomes. The additional reference-group comparison broadens the occupational perspective, although its heterogeneity limits interpretability. These comparisons provide exploratory estimates for planning more precisely characterized studies.

The small, non-probability sample and unequal group sizes are major limitations. No a priori sample-size calculation was performed, and the emergency-care group was particularly small. Non-significant results therefore cannot exclude meaningful between-group differences or demonstrate equivalence. The secondary emotional-exhaustion result arose among several unadjusted comparisons; its confidence interval was wide, and its lower limit was close to zero. The nominal statistical significance should not be interpreted as robust confirmatory evidence.

Convenience recruitment from two institutions limits generalizability and may introduce selection and non-response bias. The number of professionals invited was unavailable, preventing calculation of a participation rate. Professionals experiencing substantial distress may have been less able or willing to participate, whereas those concerned about wellbeing may have been more likely to respond. A healthy-worker or survivor effect is also possible because professionals who had left the relevant services were not represented. The direction and magnitude of these biases cannot be established from the available findings.

Self-report measures are subject to recall and social-desirability bias. Study-specific internal-consistency estimates were unavailable, limiting assessment of measurement reliability in this sample. Complete-case analysis reduced the secondary samples from 44 recruited participants to 38 for the MBI-HSS and 39 for the WHOQOL-BREF; without assessment of the missing-data mechanism, bias from incomplete responses cannot be excluded. Profession, age, experience, institution, workload, staffing, shift work, caseload, exposure to death, team functioning, and supervision may have confounded the comparisons. The small sample did not support reliable adjustment for these factors, and the cross-sectional design prevents temporal or causal interpretation.

### 4.2. Implications for Future Research

Larger multicentre studies should prespecify a limited number of primary outcomes, calculate sample size prospectively, and characterize professional roles, institutions, and working conditions in detail. Reporting should include study-specific reliability estimates, transparent missing-data procedures, effect sizes, and confidence intervals. Adequately sized samples would permit separate analyses of physicians, nurses, and other professional categories and appropriate adjustment for confounding. Longitudinal designs would help examine whether changes in workload, team resources, or exposure to suffering precede changes in emotional exhaustion and quality of life.

## 5. Conclusions

In this small exploratory sample, no statistically significant multivariate differences in burnout or quality of life were detected among oncology palliative care, intensive care, and emergency care professionals. These findings do not establish similarity or equivalence between settings. A secondary, unadjusted comparison identified a nominally significant difference in emotional exhaustion between oncology palliative care and a heterogeneous reference group. This result is hypothesis-generating and cannot be attributed to clinical setting independently of professional composition or working conditions. Replication in larger, well-characterized multicentre samples is needed before drawing setting-specific conclusions.

## Figures and Tables

**Table 1:** Sociodemographic and professional characteristics of the study groups.

Characteristic	Oncology Palliative Care *N* = 22	Intensive Care *N* = 15	Emergency Care *N* = 9	Reference Group *N* = 22
Age, years, mean (SD), valid *n*	36.91 (9.58), 22	34.47 (4.72), 15	42.63 (11.56), 8	34.61 (7.85), 22
Female, *n*/valid *n* (%)	16/22 (72.7)	9/14 (64.3)	5/9 (55.6)	18/22 (81.8)
Married/cohabiting, *n*/valid *n* (%)	14/22 (63.6)	11/14 (78.6)	6/8 (75.0)	13/22 (59.1)
Physician, *n*/valid *n* (%)	4/21 (19.0)	2/14 (14.3)	4/9 (44.4)	7/22 (31.8)
Nurse, *n*/valid *n* (%)	17/21 (81.0)	12/14 (85.7)	5/9 (55.6)	15/22 (68.2)

*N* = total group size; *n* = valid responses; SD = standard deviation. Counts and percentages use the valid-response denominators shown. Professional category was missing for one oncology palliative care participant and one intensive care participant. A dash indicates that professional-category information for the reference group is not presented; it does not denote zero participants.

**Table 2:** MBI-HSS scores across the three clinical settings.

MBI-HSS Dimension	OPC Mean (SE) [95% CI]	ICU Mean (SE) [95% CI]	EC Mean (SE) [95% CI]	F (2, 43)	*p*	Partial η^2^
Emotional exhaustion	19.77 (1.61) [16.52–23.02]	15.40 (1.95) [11.47–19.33]	15.00 (2.52) [9.92–20.08]	2.062	0.140	0.088
Personal accomplishment	36.41 (1.31) [33.77–39.05]	33.60 (1.58) [30.41–36.79]	34.33 (2.04) [30.22–38.44]	1.023	0.368	0.045
Depersonalization	5.18 (1.25) [2.66–7.70]	5.20 (1.51) [2.15–8.25]	6.00 (1.95) [2.07–9.93]	0.070	0.933	0.003

OPC = oncology palliative care; ICU = intensive care; EC = emergency care; CI = confidence interval; MBI-HSS = Maslach Burnout Inventory—Human Services Survey; SE = standard error. Higher personal accomplishment indicates a more favourable outcome. The 95% CIs were calculated from the reported SEs using residual df = 43. Tests and CIs are unadjusted for multiple comparisons.

**Table 3:** WHOQOL-BREF scores across the three clinical settings.

WHOQOL-BREF Outcome	OPC Mean (SE) [95% CI]	ICU Mean (SE) [95% CI]	EC Mean (SE) [95% CI]	F (2, 42)	*p*	Partial η^2^
Physical health	79.38 (2.55) [74.23–84.53]	81.63 (3.20) [75.17–88.09]	78.57 (3.99) [70.52–86.62]	0.223	0.801	0.011
Psychological	76.33 (3.10) [70.07–82.59]	80.36 (3.88) [72.53–88.19]	76.85 (4.84) [67.08–86.62]	0.348	0.708	0.016
Social relationships	78.03 (3.30) [71.37–84.69]	75.60 (4.13) [67.27–83.93]	78.70 (5.15) [68.31–89.09]	0.146	0.865	0.007
Environment	71.02 (2.43) [66.12–75.92]	69.87 (3.04) [63.74–76.00]	73.61 (3.80) [65.94–81.28]	0.300	0.742	0.014
General QoL/health	72.16 (2.82) [66.47–77.85]	77.68 (3.54) [70.54–84.82]	76.39 (4.41) [67.49–85.29]	0.833	0.442	0.038

OPC = oncology palliative care; ICU = intensive care; EC = emergency care; CI = confidence interval; SE = standard error. Higher scores indicate better perceived quality of life. General QoL/health denotes the combined outcome from the two general items and is distinct from the four domains. The 95% CIs were calculated from the reported SEs using residual df = 42. Tests and CIs are unadjusted for multiple comparisons.

**Table 4:** Secondary MBI-HSS comparison between oncology palliative care and the heterogeneous reference group.

MBI-HSS Dimension	OPC Mean (SE)	REF Mean (SE)	Mean Diff.	95% CI	F (1, 36)	*p*	Partial η^2^
Emotional exhaustion	19.75 (1.58)	13.97 (1.85)	5.78	0.37–11.19	4.699	0.037	0.115
Personal accomplishment	36.77 (1.76)	31.59 (2.05)	5.18	−0.81–11.17	3.079	0.088	0.079
Depersonalization	5.04 (1.23)	6.19 (1.49)	−1.15	−5.50–3.20	0.287	0.595	0.008

OPC = oncology palliative care; REF = heterogeneous reference group; CI = confidence interval; MBI-HSS = Maslach Burnout Inventory—Human Services Survey; SE = standard error. Mean differences are OPC minus REF. The multivariate analysis included *n* = 38 complete cases out of *N* = 44 recruited participants in the two groups combined. Tests and CIs are unadjusted for multiple comparisons.

**Table 5:** Secondary WHOQOL-BREF comparison between oncology palliative care and the heterogeneous reference group.

WHOQOL-BREF Outcome	OPC Mean (SE)	REF Mean (SE)	Mean Diff.	95% CI	F (1, 37)	*p*	Partial η^2^
Physical health	78.34 (2.74)	74.69 (3.05)	3.65	−4.98–12.28	0.735	0.397	0.020
Psychological	75.40 (3.47)	75.90 (3.87)	−0.50	−11.83–10.83	0.008	0.929	0.000
Social relationships	76.79 (3.62)	78.37 (4.03)	−1.58	−12.97–9.81	0.079	0.780	0.002
Environment	70.38 (2.09)	66.06 (2.33)	4.32	−2.28–10.92	1.761	0.193	0.045
General QoL/health	71.43 (3.05)	71.73 (3.40)	−0.30	−9.91–9.31	0.004	0.950	0.000

OPC = oncology palliative care; REF = heterogeneous reference group; CI = confidence interval; SE = standard error. Mean differences are OPC minus REF. The multivariate analysis included *n* = 39 complete cases out of *N* = 44 recruited participants in the two groups combined. General QoL/health is the combined outcome from the two general items, not a fifth domain. Tests and CIs are unadjusted for multiple comparisons.

## Data Availability

The data are not publicly available because of ethical and confidentiality restrictions. Anonymized data may be available from the corresponding author on reasonable request, subject to the relevant ethical and institutional approvals.

## Supplementary Materials

The following supporting information can be downloaded at https://www.mdpi.com/article/10.3390/healthcare14193239/s1, File: STROBE Statement—Checklist for Cross-Sectional Studies.
