# Supplementary material for: Burnout and Quality of Life Among Oncology Palliative Care Professionals: An Exploratory Study
Source: Healthcare (Basel). 2026 Oct 1;14(19):3239. doi: 10.3390/healthcare14193239 (PMC13664855; doi:10.3390/healthcare14193239)
Supplement: Supplementary file 1 [file healthcare-14-03239-s001.zip › healthcare-4533883-supplementary.pdf]

STROBE Statement—Checklist for Cross-Sectional Studies

Manuscript: ***Burnout and Quality of Life among Oncology Palliative Care Professionals: An Exploratory Study***

The checklist identifies where each STROBE item is addressed in the revised manuscript. Items not applicable to this exploratory cross-sectional design are marked N/A.

Item	STROBE reporting recommendation	Where addressed in the revised manuscript
**1a**	Indicate the study design using a commonly used term in the title or abstract.	Title and Abstract, pp. 1-2
**1b**	Provide an informative and balanced summary of what was done and what was found.	Abstract, pp. 1-2
**2**	Explain the scientific background and rationale for the investigation.	Introduction, pp. 2-3
**3**	State the specific objectives, including any prespecified hypotheses.	Introduction, p. 3 - primary and secondary exploratory objectives; no confirmatory hypothesis is claimed.
**4**	Present the key elements of the study design early in the paper.	Methods, Section 2.1, p. 3
**5**	Describe the setting, locations, and relevant periods of recruitment, exposure, follow-up, and data collection.	Methods, Sections 2.1-2.3, pp. 3-4 - two hospital units within the same Portuguese public hospital organization; data were collected between December 2025 and February 2026 using a self-administered assessment protocol.
**6a**	Give eligibility criteria and the sources and methods of participant selection.	Methods, Section 2.2, pp. 3-4 - adult healthcare professionals; convenience sampling; voluntary participation and written informed consent.
**7**	Clearly define outcomes, exposures, predictors, potential confounders, and effect modifiers, as applicable.	Methods, Sections 2.4-2.5, pp. 4-5; Discussion, pp. 9-11 - burnout and quality-of-life outcomes are defined; potential confounding by profession, age, institution, and working conditions is acknowledged.
**8**	For each variable of interest, describe data sources and measurement methods, and indicate comparability across groups.	Methods, Sections 2.4.1-2.4.3, p. 4 - sociodemographic/professional questionnaire, MBI-HSS, and WHOQOL-BREF; instruments were applied across study groups.
**9**	Describe efforts to address potential sources of bias.	Methods, Section 2.3, p. 4; Discussion, Section 4.1, pp. 10-11 - standardized self-administered protocol, confidentiality/anonymity, and discussion of selection, non-response, self-report, healthy-worker/survivor, and confounding biases.
**10**	Explain how the study size was determined.	Methods, Section 2.5, pp. 4-5 - no a priori sample-size calculation; the small, unequal convenience sample is explicitly treated as exploratory.
**11**	Explain how quantitative variables were handled in the analyses, including any groupings or transformations.	Methods, Sections 2.4.2-2.5, pp. 4-5 - MBI-HSS dimensions analysed continuously; WHOQOL-BREF scores transformed to 0-100; burnout categories were not used.
**12a**	Describe all statistical methods, including methods used to control for confounding.	Methods, Section 2.5, p. 5 - MANOVA, Pillai's Trace/Hotelling's Trace, univariate tests, 95% CIs, exact p-values, partial eta-squared, alpha level, and multiplicity statement. Comparisons are explicitly unadjusted.
**12b**	Describe methods used to examine subgroups and interactions.	N/A - no formal subgroup or interaction analyses were performed. Primary and secondary group comparisons are reported separately.
**12c**	Explain how missing data were addressed.	Methods, Section 2.6, p. 5 - available-case descriptive statistics and complete-case multivariate analyses; no imputation of missing data was performed.
**12d**	If applicable, describe analytical methods that account for the sampling strategy.	N/A - non-probability convenience sampling; no complex survey design or weighting was used.

Item	STROBE reporting recommendation	Where addressed in the revised manuscript
**12e**	Describe any sensitivity analyses.	N/A - no formal sensitivity analyses were included; interpretation emphasizes effect sizes, confidence intervals, and uncertainty in this exploratory sample.
**13a**	Report the number of individuals at each relevant stage of the study, including numbers analysed.	Results, Section 3.1, pp. 5-6; Sections 3.2-3.4 and Tables 1-5, pp. 6-8 - N=68, subgroup totals, valid-response denominators, and analysis-specific complete-case n are reported.
**13b**	Give reasons for non-participation at each stage, where applicable.	N/A to the reported recruitment flow - convenience recruitment did not use a closed sampling frame for the entire eligible workforce; no formal participation rate is presented.
**13c**	Consider use of a flow diagram.	Not included - participant totals and analysis-specific denominators are reported directly in the text and tables.
**14a**	Provide characteristics of study participants and information on relevant exposures and potential confounders.	Results, Section 3.1 and Table 1, pp. 5-6 - age, sex, marital status, professional category, and study group are reported; the heterogeneous reference group comprised 15 nurses and 7 physicians.
**14b**	Indicate the number of participants with missing data for each variable of interest.	Methods, Section 2.6, p. 5; Results, Section 3.1 and Table 1, pp. 5-6; Sections 3.4.1-3.4.2, pp. 7-8.
**15**	Report outcome data or summary measures.	Results, Tables 2-5, pp. 6-8 - burnout and quality-of-life estimates by comparison group.
**16a**	Report unadjusted estimates and their precision; report adjusted estimates if applicable and identify adjusted confounders.	Results, Sections 3.2-3.4 and Tables 2-5, pp. 6-8 - means, mean differences where applicable, 95% CIs, exact p-values, and partial eta-squared. No adjusted model is claimed.
**16b**	If continuous variables were categorized, report category boundaries.	N/A - principal MBI-HSS and WHOQOL-BREF outcomes were analysed as continuous variables.
**16c**	If relevant, translate relative risk estimates into absolute risk for a meaningful time period.	N/A - no relative risk estimates were calculated.
**17**	Report other analyses performed, such as subgroup, interaction, or sensitivity analyses.	Results, Section 3.4, pp. 7-8 - prespecified secondary comparison with the heterogeneous reference group. No formal subgroup, interaction, or sensitivity analyses were performed.
**18**	Summarize key results with reference to the study objectives.	Discussion, p. 9; Conclusions, p. 11 - primary non-significant multivariate comparisons are distinguished from the secondary hypothesis-generating emotional-exhaustion finding.
**19**	Discuss study limitations, including potential sources of bias or imprecision and their likely direction or magnitude.	Discussion, Section 4.1, pp. 10-11 - small and unequal groups, missing data, multiple testing, convenience recruitment, selection/non-response bias, self-report bias, residual confounding, and cross-sectional design.
**20**	Provide a cautious overall interpretation considering objectives, limitations, multiplicity, related evidence, and other relevant findings.	Discussion, pp. 9-11 - interpretation remains exploratory; non-significance is not interpreted as equivalence and the secondary finding is treated as hypothesis-generating.
**21**	Discuss the generalisability (external validity) of the findings.	Discussion, Section 4.1, pp. 10-11 - small convenience sample from two hospital units and limited occupational characterization are acknowledged as constraints on generalisability.
**22**	State the source of funding and the role of funders, if applicable.	Funding statement, p. 11 - no external funding.
